# The Use of Next Generation Sequencing to Detect Early Implant Infections: The Future of Microbiology and Targeted Antibiotic Treatment

**DOI:** 10.1093/asjof/ojae007.077

**Published:** 2024-04-12

**Authors:** Jaime L Bernstein, Anna Vaeth, Karina Condez, Kristen Castellano, Grant Black, David Otterburn

**Affiliations:** NewYork-Presbyterian -Cornell/Columbia, New York, NY; Weill Cornell Medical College, New York; Weill Cornell Medical College, New York; Weill Cornell Medical College, New York; Weill Cornell Medical College, New York; Weill Cornell Medical College, New York

## Abstract

**Goals/Purpose:**

The presence of fluid around foreign bodies, particularly breast implants, is a well-established phenomenon; However scant data exists regarding the composition of this fluid. The difficulty in studying this fluid arises from the lack of safe access to aspirate fluid post-breast augmentation, and by the loss of sterility once fluid exits the body via a drain bulb as traditionally placed during breast reconstruction. Our innovative breast reconstruction approach utilizes a drainless technique with a dual chamber tissue expander (TE) in the pre-pectoral plane, offering a unique avenue to sterilely aspirate peri-implant fluid through a percutaneous drainage port. In this study, we employ Next Generation Sequencing (NGS), utilizing polymerase chain reaction technology, to discern the composition of microbial DNA within peri-prosthetic breast implant fluid. NGS, known for its precision and cost-effectiveness, circumvents the biases inherent in traditional culture methods. By integrating this cutting-edge technology with our breast reconstruction methodology, we sought to study peri-prosthetic breast implant fluid to better understand when implant infections arise and if peri-prosthetic fluid could detect infections before clinical symptoms arise.

**Methods/Technique:**

This is a single surgeon, prospective study of patients undergoing mastectomy, followed by pre-pectoral, drainless, TE reconstruction. Peri-prosthetic fluid was collected through the TE drainage port in the OR immediately after closure, at 1 week post op, and 3 weeks post op. This fluid was sent for traditional culture and NGS. Patients were observed for signs of infection, antibiotics, return to OR, or implant loss.

**Results/Complications:**

33 breasts from 20 patients were included. Patients had a mean age of 50 years. The average length of drainage was 21 days post-operative, with a mean of 131 cc of fluid drained per breast per week. Eight breasts had concern for skin necrosis. In 25 breasts (76%), complete sterility was achieved at the end of the case with no NGS detection of microorganisms in the initial peri-implant fluid sample. However, NGS detected microorganisms in the initial sample in 8 breasts (24%). In 5 of these 8 breasts, microorganisms were cleared by the 1-week sample and another 2 cleared by 3 weeks. The remaining 1 breast continued to have positive NGS and developed clinical signs of infections by week 2, which were not picked up by traditional culture until week 3. One breast with skin necrosis developed a positive NGS at week 3 and clinical signs of infection at week 4. No other clinical infections were seen throughout the study.

**Conclusion:**

We are one of the first groups to demonstrate the ability to achieve peri-implant sterility at the time of implant insertion in the operating room. This could have significant implications for understanding the necessity of peri-operative antibiotics following breast implant placement. Our investigation into the microbial composition of ostensibly "sterile" peri-prosthetic breast implant fluid provides insight into when microorganisms arise and when they become clinical infections. These promising preliminary results suggests that peri-prosthetic fluid monitoring through Next-Generation Sequencing (NGS) holds significant potential in preemptively detecting implant infections before the onset of clinical symptoms, thereby reducing infection rates. Furthermore, our findings open avenues for targeted antibiotic therapy, contributing to the imperative goal of antibiotic stewardship. Ongoing work is beginning to explore the impact of microorganisms within the fluid on breast capsule formation, as we strive to enhance our understanding of breast implants and infections.

